# A comparative study of seasonal irrigation demand and actual water supply in the Kaleshwaram project command area

**DOI:** 10.1038/s41598-026-56847-1

**Published:** 2026-07-02

**Authors:** Rahul Karra, Majji Kiranmai Reddy

**Affiliations:** Department of Life Sciences, Gandhi Institute of Technology and Management (Deemed to be University), Vishakhapatnam-530045, Andhra Pradesh India

**Keywords:** Kaleshwaram lift irrigation scheme, KLIS command area, Irrigation demand, Water supply, Seasonal water deficit, Monthly water balance, CROPWAT, SWAT, GIS, Canal scheduling, Climate sciences, Environmental sciences, Hydrology

## Abstract

This paper assesses seasonal and spatial mismatch between irrigation water demands and water supply in the Kaleshwaram Lift Irrigation Scheme (KLIS) command area, India where inflows dependent on the monsoon seasons, high evapotranspiration, timing of canal-release, and planting schemes have a combined effect on irrigation reliability. Based on multi-year occurrences data, which covers climate-data between 2000 and 2023, groundwater-observations data between 2015 and 2023, reservoir and canal-operation data between 2019 and 2023, and crops/land-use data between 2022 and 2023, the study generates a monthly water-balance framework, here variables specific periods were used according to the verified data availability. Digital Elevation Model (DEM)-based Geographic Information System (GIS) preprocessing was combined with the results of CROPWAT and Soil and Water Assessment Tool (SWAT) to estimate the reference evapotranspiration (ET 0), crop evapotranspiration (ET c), effective rainfall, runoff, recharge, and soil-moisture change. The available supply was estimated by using an addition of canal release, reservoir- operation values, ground water contribution, and contribution of return-flow. These findings indicate that irrigation takes up the largest portion of the annual water needs with about 98.4% of total yearly needs i.e. 1650 Mm^3^ of the total 1677 Mm^3^. The gross supply available was around 730 Mm^3^ which could only satisfy about 43.5% of the overall demand leaving behind an annual unfulfilled demand of around 947 Mm^3^, which would cater to almost 56.5% of the aggregate demand. The greatest demand was at April-May where it was approximately 197–212 Mm^3^/month, or 11.8–12.7% annual demand during this period. None of the months had over supply and highest monthly loss was in the months of March–May corresponding to a range of approximately 102–127 Mm^3^ monthly which only constituted about 10.8–13.4% of the annual loss in that year. The seasonal interpretation shows that the periods of water-stress are peak three months pre-monsoon and Rabi (spring) and Kharif (autumn) depend heavily on storage of the reservoir, efficient rainfall distribution, and timing of the canals. The results prove that annual supply volumes cannot be considered the only factors of KLIS performance, monthly alignment of crop-water demand and monsoon recharge with the performance of the storage and the canal delivery plays a vital role. The research offers application evidence on how to better reservoir rule curves, canal-sort, conjunctive ground water utilization, crop-arrangement and deficit-irrigation techniques in monsoon-based command regions that are semi-arid.

## Introduction

Water is a basic socio-economic resource, and the foundation of food security and human well-being. The growth in population, the change in land-use and the variation in climate are, however, weakening its consistent supply. These stressors have led to the over-abstraction of groundwater, decrease in streamflow, and increasing inter-sectoral competition in most areas- the impact of unsustainable abstractions, inadequately coordinated allocation changes and the lack of knowledge of the basin-scale hydrology, all of which impair the development of the long term and the physical condition of ecosystems^1^. Water resource management as a sustainable phenomenon is thus a challenge to planners to quantify and balance the supply and demand of water to the right spatial and temporal scales.

These problems are particularly severe in the dry and semi-arid areas, where there is low precipitation and extremely irregular, as well as there is high evaporative demand that makes shortage of water more likely. The same situation is observed in Mexico: almost half of the national territory is arid or semi-arid, and water resources are highly limited by a low level of rainfall and a high rate of evapotranspiration^2,3^. Due to their similar characteristics, semi-arid and Mediterranean-type basins are found globally, and agriculture is commonly the predominant sector of water use alongside the economy commonly being highly reliant on irrigation of high-value products like vineyards and horticultural produce^4,5^. In such conditions, the water supply of renewable water is often less than their demand in irrigation, which results in the chronic water insufficiency, overexploitation of aquifers, and the conflict between the users of the water.

The effects of climate change and climate variability exacerbate such pressures. The change in the timing of rainfall, its intensity, increase in temperature and increasing evaporative demand of atmosphere change the seasonal runoff generation, soil moisture recharge, and ground water replenishment. Both regional and global evaluations show that although the majority of regions are not experiencing major changes in annual precipitation, increasing evapotranspiration (ET) may significantly augment water demands on irrigation and escalate the likelihood of drought in water-challenged environments^6–8^. Climate research further solidifies that the trends have already dampened productivity growth in the world, and current irrigation systems are becoming less sustainable unless water-use efficiency increases and crop patterns and irrigation timelines are changed^9–11^.

In this situation, the massive irrigations in semi-arid monsoon areas have two-fold degrees of operational problems. On the one hand, they have been meant to stabilize agricultural production by absorbing intra-annual fluctuations in hydrology by reservoirs, canal systems and lift irrigation systems. Conversely, they are limited in performing due to their small storage capacity, extremely seasonal inflows, and increasing seasonal demands on crop-water. The lift-irrigation projects of the meandering rivers in South Asia need to mix and match the strong monsoon inflows, achieved during the Kharif season (June–October) with the limited inter-seasonal reservoirs, the cultivation requirements of the Rabi season (November–March) and the Pre-monsoon crop production cycle (April–May), and the domestic needs of the rural and peri-urban settlement. The ability to match seasonal irrigation needs with the real water flows received in the form of canal irrigation, underground water and ground water return is vital to the long-term viability of such projects.

This functional issue requires operationally based water-balance models that can transform, at monthly and seasonal levels, the way rainfall, ET, runoff, recharge, and soil water respond into water supply and water demand in crops. Current improvements on soil-vegetation-atmosphere transfer (SVAT) models and watershed-scale models such as the Soil and Water Assessment Tool (SWAT) are now providing an opportunity to make quantitative approximations of the main fluxes: reference ET, crop evapotranspiration, effective rainfall and soil-moisture processes in even data-limited basins, allowing structured comparisons of supply and demand^12–14^.

However, the supply-demand literature available in India and other semi-arid areas has assumed irrigation needs analysis with generic SWAT or CROPWAT models or some district-level estimates of needs without directly relating such models or estimates to the practical behaviour of large-scale lift-irrigation systems with seasonal variations in hydrology. Most Indian irrigation literature records net irrigation deficits or annualised irrigation demands but does not often quantify the disconnect between supply and demand on sub-annual basis, during the most crucial non-monsoon seasons, Rabi (November–March) and in thepre-monsoon (April–May), i.e. when storage is minimal, inflows are low, and crop water demand is high. Substantial literature gap therefore exists in the ability of large, multi-stage lift irrigation projects to maintain command-area delivery in increasing evaporation and separative demands and increasing uncertainty in monsoon-driven inflows. This difference is substantial since the functioning of irrigation systems in semi-arid monsoon climates is not an annual but a seasonal event: the interaction of supply, storage, and demand, month in month out, could, and must, be specifically clarified, a step in the analysis which earlier investigations have not undertaken satisfactorily.

The Kaleshwaram Lift Irrigation Scheme (KLIS), which is situated in the basin of Godavari in India will offer a critical case-study in this broader context. Being one of the largest current semi-arid irrigation projects that constitute several barrages, reservoirs and lift stages to provide irrigation and domestic water supplies on a massive command area, KLIS is set to enable guaranteed water provision. However, its sustainability performance relies on the ability to fulfil the monthly and seasonal water needs of the crop under existing monsoon hydrological conditions and rising atmospheric evaporative stresses as a result of the growing crop areas imposed by the current cropping systems.

The current research fits right into this gap by a physically based, monthly water-balance model that considers actual KLIS operational supply data and a quantitative evaluation of irrigation demand and the disconnect of supply and delivered volume seasonally. The use of SWAT and CROPWAT models,  in the present analysis has solved the month-to-month interaction of the supply, storage and demand at the system level, in fact this presents the first evidence-based quantification of the seasonal water shortage in the KLIS command area. In particular, this paper: (i) determines rainfall, reference evapotranspiration, crop water requirements, and soil-moisture dynamics in a physically consistent monthly water-balance framework; (ii) determines such fluxes at the command scale in relation to the Kharif (June–October), Rabi (November–March), and Pre-monsoon (April–May) periods; (iii) explicitly separates irrigation and The results produce evidence-based advice on how to run a reservoir, when to plan a canal, how best to optimise cropping patterns, and use conjunctive ground water in extensive semi-arid irrigation projects.

## Literature review

### Water-balance modelling in semi-arid and monsoon-dominated systems

Water-balance analysis is a well-established method to measure partitioning of precipitation into evapotranspiration, runoff, recharge, and storage change either of which combine to regulate water availability in any basin^1,15,16^. This partitioning is especially sensitive to rainfall scenarios in semi-arid areas: even slight shifts in precipitation levels may create imbalanced amounts of available blue water, as is the case in arid basins where high evaporative consumption acts to absorb most of the incoming rainfall^3^. The difficulty is increased by the climates, which are dominated by monsoons, where more than 70–80% of total yearly rainfall can be packed in just four to five months reducing recharge and generation of runoffs to a tiny span, leaving the remaining months highly reliant on stored water^17^.

This seasonal precipitation has been found to be the main structural factor behind the stress of irrigation water, especially in the Indian situation. Using the Soil and Water Assessment Tool (SWAT) on 12 large Indian river basins,^17^ showed that in the even moderate warming climate, water availability reduces significantly during the Rabi-season (November–March) as the soil moisture reduces, because soils become drier due to increased evapotranspiration and diminished base flows. On the same note^18^ used satellite-based ET to calibrate a distributed hydrological model of Indian irrigation systems and found that central to the issue of large canal commands is the seasonal disconnect between monsoon-driven recharge and the seasonal demand in the dry season. The results directly apply to KLIS that has a basis on the monsoon inflows collected during Kharif (June-October) periods to maintain irrigation during Rabi and pre-monsoon seasons when natural inflows are low.

### Canal command area performance in India: empirical evidence

The canal-based irrigation systems have been subject of a large amount of India-specific research, which has invariably shown discrepancies in the operational performance of the systems as intended by their design or as delivered. By the year 1999^19^ had developed the fact that most of the major and medium irrigation projects in India are functioning way below their design irrigation intensity, contributing the underperformance of the projects to a mixture of sedimentation effect, conveyance losses and fail-matching seasonal release schedules. Dhawan^20^ also showed that in places where the physical infrastructure is operational, lack of demand-side analysis at the command scale in the context of canal releases makes the canal releases to be unlinked to real crop water needs and as a result of this, there was simultaneous surplus in head-reaches and constant shortfall in tail-reaches.

These findings have been supported by more recent empirical data on peninsular India foreshadowed by hydro-climatic region of direct interest to KLIS. The study by^21^ focused on groundwater depletion in the Krishna Godavari delta basin and provided evidence that farmers gradually turn to compensating the subject water with the groundwater to respond to the unreliable and seasonally misgrained canal releases to the region, which causes an accelerating decrease in the aquifers during the Rabi and the pre-monsoon seasons. This practice of conjunctive use, where increasing supply is necessitated by the unreliability of supply but not by careful management, is reflective of the situation observed in the KLIS supply area in which groundwater supplementation in non-monsoon months is prevalent but disorganized (CWC, 2021). Shah^22^ also found that the deployment of canal water delivery hours based on the average hydrological conditions are always unprepared during the years of below-average monsoon conditions, revealing the insufficiency of fixed operation regulations when they must work with seasonal changes.

At project level^23^ tested conjunctive use in the Maharashtra canal commands and concluded that in the absence of a monthly-resolution demand-supply accounting framework, the water managers had no information by which to predict Rabi-season shortages and pre-position groundwater supplies. This was further analysed by Kumar et al.^24^, who found that in Indian irrigation systems water productivity is increasing but mostly due to improvements in scheduling of the seasons and estimating the demands rather than due to the expansion of infrastructure. The present study adopts a water-balance method every month directly inspired by these findings.

### SWAT and process-based modelling: methodological relevance

SWAT model has found extensive applications in the Indian river basins to simulate water-balance with a physically consistent model to predict reference evapotranspiration, effective rainfall, runoff and soil-moisture interactions of heterogeneous agricultural landscapes^12,14^. Its ability to map large, irrigated commands (where cropping patterns change spatially between Kharif and Rabi seasons), basins by using discrete units of hydrological response, each with exclusive soil, land-use, and management properties, gives it a particular application in large scale irrigated commands. Its applicability to Indian basins through data limitations was demonstrated by^17^, and later research revealed that under suitable parameterisation of auto-irrigation, SWAT can be used to realistically simulate the timing of irrigation and soil-moisture responses in response to varying management regimes^25,26^.

Nevertheless, one similarity throughout the SWAT literature is that the model results are commonly reported in year or event rates, and the operational supply-demand analysis at the command level (disaggregated by month and season) is seldom done^13^. The majority of the applications cease at the estimation of the irrigation demand excluding actual data of the supply through the reservoir releases and return flows. It fills this methodological void by placing SWAT-based ET plus crop-demand estimates into a monthly water-balance model that expressly involves observed KLIS data on supply.

### Climate change, evapotranspiration trends, and irrigation demand

Regional and worldwide evaluation indicates that the growing evaporative pressure of the atmosphere is steadily raising irrigation water demands in semi-arid regions both regardless of changes in precipitation ^6–8^. With regards to India in particular, projections in CMIP5 and CMIP6 describe scenarios whereby reference evapotranspiration in the Deccan Plateau (where KLIS is located) is going to rise by 5–12% by mid-century, with the largest gains during the pre-monsoon of April-May when crop water needs are already at their peak^6^. Ortiz-Bobea et al.^11^ affirm that the trends have already stifled at productivity growth in rain and irrigated agriculture, and^9^ stress that the instruments of adaptation strategies should focus more on better timing of irrigation and water accounting at the level of seasonal resolution.

These climatic stresses are exacerbating the already structural weakness of large lift-irrigation schemes. This approach to water allocation, based on the assumptions of the long-term average supply, is becoming insufficient in non-stationary climate conditions, and instead of it, the decision-support framework that tackles the seasonal nature of the climate drive on demand and infrastructure-constrained supply at a monthly level is needed, which is, in fact, the analytical goal of the current research.

### Research gaps and positioning of the present study

Combined, the literature shows that there are three gaps that converge and which the current study is placed to fill. First, the methodology of water-balance modelling in semi-arid and monsoon-based basins is well-established but, secondly, very few have explicitly implemented a monthly-resolution supply-demand model in regard to a large-scale lift-irrigation command in India, where inter-seasonal storage in relation to monsoon-season inflow capture, storage, and Rabi season crop demand is central to operations Second, despite uniform success in using the seasonal timing mismatch underlying the chronic deficit conditions to establish the location has been demonstrated in India specific canal irrigation studies, these studies have not been based on physically grounded models of water-balance but instead on observational, or survey-based studies. Third, the KLIS command area, although one of the largest and most recently developed lift -irrigation projects in India, has not undergone a systematic, evidence-based supply-demand analysis on a monthly and seasonal level.

The current research paper bridges these gaps by combining a monthly water-balance model with real KLIS operating supply data, crop water demand modeling, and clear distinction of irrigation and domestic needs in each of the Kharif (June to October), Rabi (November to March) and pre-monsoon (April to May) seasons. By so doing, it answers directly the calls in the Indian irrigation literature, to quantitative, seasonally resolved measures of performance that can be used in reservoir operation, canal scheduling, and conjunctive management of groundwater in large semi-arid irrigation systems.

## Methodology

**Fig. 1:**
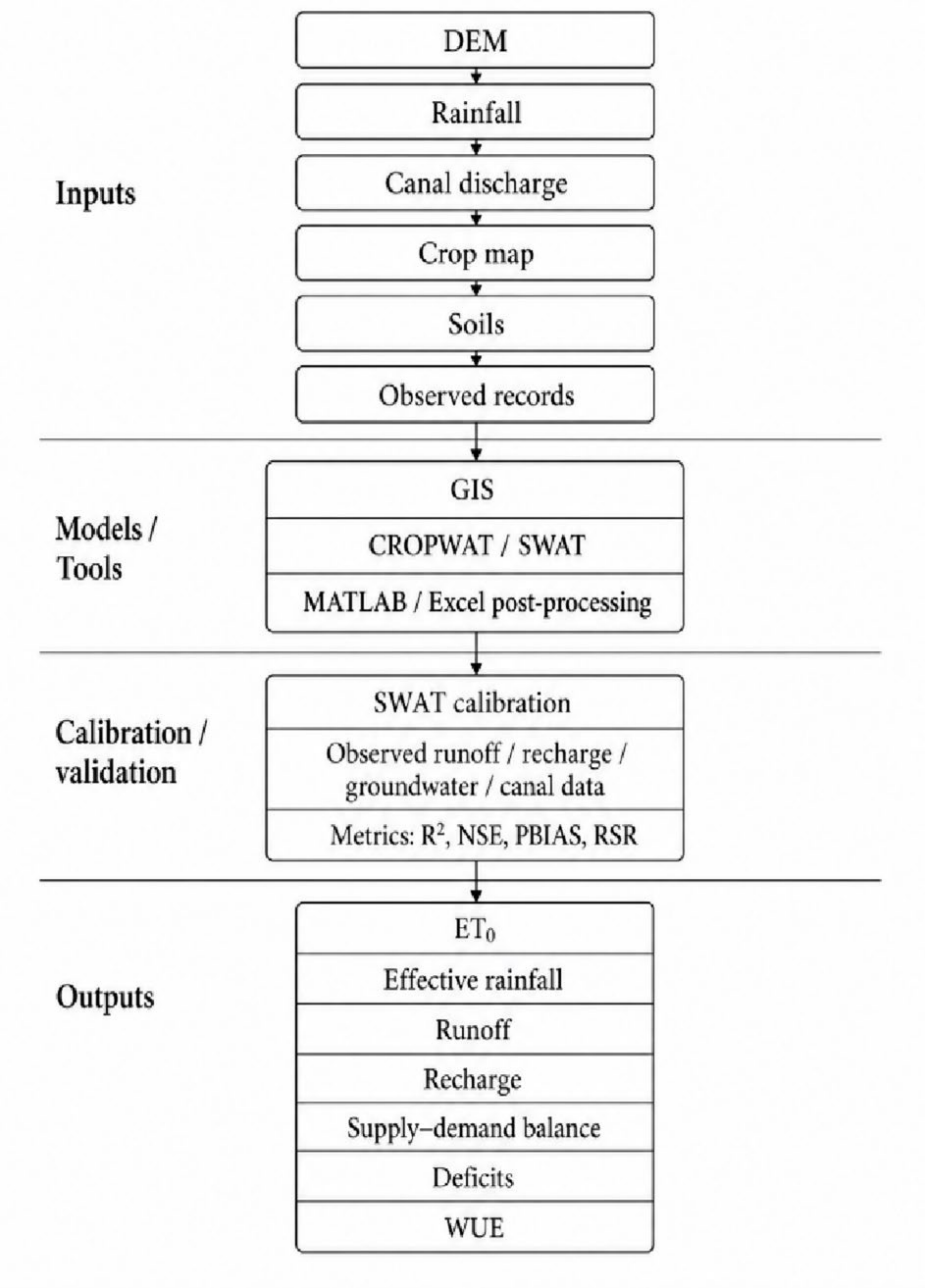
Conceptual methodology framework for the KLIS irrigation demand–supply assessment, integrating SWAT 2012 hydrological modelling (calibration NSE = 0.76, R^2^ = 0.81), CROPWAT 8.0 crop-water estimation, GIS-based spatial pre-processing (ArcGIS 10.8.2), and observed supply data integration to produce monthly and seasonal water-balance and deficit outputs across Kharif (June–October), Rabi (November–March), and pre-monsoon (April–May) seasons.

This paper combines the hydrological modelling of the system with Geographic Information System (GIS)-spatial analysis and the estimation of the irrigation water demand and the actual water supply of the Kaleshwaram Lift Irrigation Scheme (KLIS) command area in Telangana, India. Figure 1 shows the integrated modelling workflow. Every input to the models, calibration-validation processes and uncertainty assessments are recorded to ensure that they are scientifically reproducible.

### Study area description

**Fig. 2:**
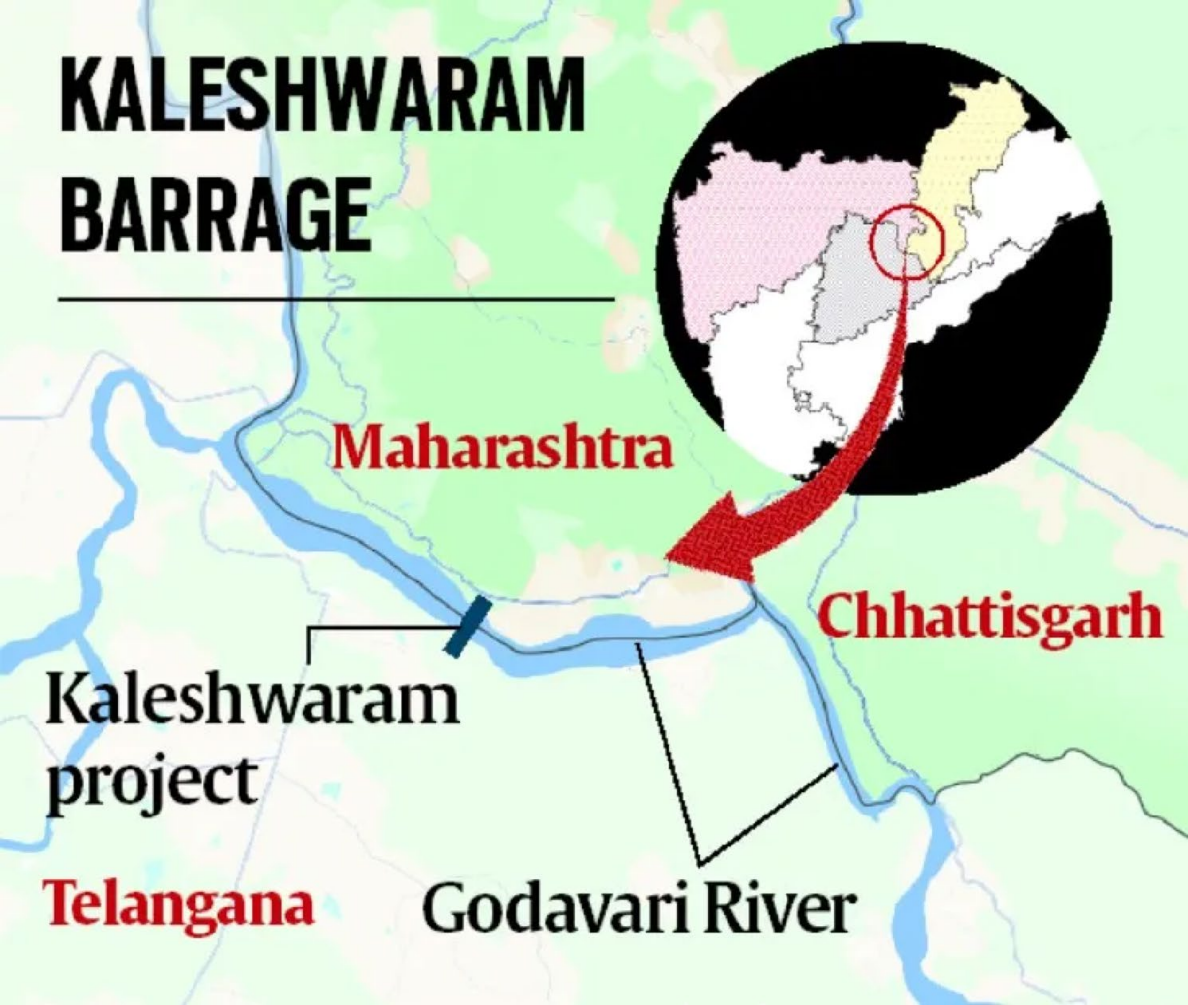
KLIS command area spatial extent showing the 17 delineated sub-basins (SB-01 to SB-17), Left Main Canal network, branch canals, three principal barrages (Medigadda, Annaram, Sundilla), and CWC streamflow gauging stations used for SWAT calibration and validation. Sub-basins delineated from SRTM 30-m DEM using ArcGIS 10.8.2/ArcSWAT 2012. Total command area ≈ 7620 km^2^.

The KLIS command area is in the north-central Telangana state, India, between latitudes 17° 30′ N and 19° 00′ N and longitudes 78° 30′ E and 80° 00′ E, in the lower sub-basin of Godavari and Maner rivers (Fig. 2). The total area of delineated command is about 7620 km^2^ (18.82 lakh acres), that includes the Left Main Canal (LMC) and Right Main Canal (RMC) command zones in 13 districts in Telangana. This large command extent supports the use of area average values for command scale water balance estimation.  KLIS would provide 147 TMC (thousand million cubic feet) of water on three large barrages, including Medigadda, Annaram, and Sundilla, and balancing reservoirs (GoT, 2019; CWC, 2021). A semi-arid and monsoonal climate defines the command area with an average annual rainfall of 900–1050 mm of which more than 80% is received during the Kharif rainy season. The prevailing soils are Vertisols and Alfisols, and the major system of crop rotation includes paddy (Kharif), cotton and pulses (Rabi) and vegetables and sunflower (pre-monsoon) .

### Data sources

Table 1 summarises all primary and secondary input datasets with spatial/temporal resolution, period of record, and data source.

**Table 1:** Summary of input datasets.

Dataset	Resolution/scale	Period	Source
Digital elevation model (DEM)	30 m (SRTM)	2023	NASA/USGS SRTM (earthexplorer.usgs.gov)
Climate data	Daily station records	2000–2023	India Meteorological Department (IMD); CWC gauge stations
Crop coefficients (Kc) & calendars	FAO-56; field-adjusted	2022–23	Allen et al.^27^; KLIS agronomy reports; field surveys
Land use/cropping pattern	30 m raster + vector polygons	2022–23	NRSC Bhuvan LULC; TRSAC, Telangana
Soil data	1:50,000 soil maps	2022	NBSS&LUP, Nagpur
Canal network	Georeferenced vector layers	Current	KLIS Project Authority, Irrigation & CAD Dept., GoT (2021)
Command boundary	Vector polygon	Current	KLIS Project Authority; Survey of India toposheet
Reservoir and storage data	Tabular + CAD drawings	2019–2023	CWC Reservoir Regulation Reports; Irrigation & CAD Dept., GoT
Groundwater levels	Observation wells (quarterly)	2015–2023	Telangana State GW Dept.; CGWB district reports
Domestic water requirement	Administrative boundary scale	2023	Census of India (2011, projected); TWDB norms; GoT DPR
Streamflow (calibration/validation)	Daily gauge records	2005–2022	CWC [45] hydrological observation stations, Godavari & Maner

### DEM processing and watershed delineation

Flow direction, flow accumulation, stream network and watershed boundaries were determined using the standard D8 algorithm^28^ in ArcGIS Desktop version 10.8.2^29^ which used standard SRTM 30-m DEM. The ArcHydro toolset was used to apply sink-filling before extracting streams. ArcSWAT 2012 interface was used to define the KLIS command area into 17 sub-basins and 164 HRUs^30^ and visualised in Fig. 2.

Classification was made of the HRUs as unique combinations of land use class, soil type and slope class where threshold values of 5 (land use), 10 (soil type) and 10 (slope type) were used uniformly among all the sub-basins^31,14^. These thresholds are commonly employed in the SWAT literature, and have been confirmed by global sensitivity analyses to provide stable water-balance prediction without undue computational overhead in semi-arid agricultural catchments^32^. Simulated annual runoff and recharge through a local threshold sensitivity test (varying thresholds between 2%/5% and 10%/20%) demonstrated that the range of thresholds varied by less than 3.5% across the entire threshold range, confirming the values used in the KLIS command area.

### SWAT model setup, calibration, and validation

Each month e.g. of a 164 HrUs of surface runoff, deep percolation (recharge), lateral flow and change of soil-water storage were simulated using SWAT 2012 (Revision 700^33^). A warm up period (2000–2002) initialised model state variable. The SPAW Hydrology model was used to parameterise soil hydraulic properties based on NBSS&LUP 1:50,000 maps^34^. About NRSC Bhuvan (2022-23) images were reclassified in SWAT land-use categories.

Calibration and validation: The model was tested both on observed daily streamflow at CWC measuring stations on the Godavari (Perur) and Maner (Mahadevpur) rivers in 2003–2015, and autonomously in 2016–2022. CGWB/SGWD groundwater observation wells (24) were used as secondary calibration of the estimates of recharge. The parameters sensitivity was evaluated using SWAT-CUP v5.3.7^35^] through SUFI-2 algorithm in 1000 simulation runs. Table 2 shows the top ten sensitive parameters and fitted values.

**Table 2:** SWAT calibration parameters and fitted values.

Parameter	Description	Calibration Range	Fitted Value
CN2	SCS Curve Number for moisture condition II (relative)	−35% to + 35%	−14%
ALPHA_BF	Baseflow recession constant (days⁻¹)	0.01–0.99	0.042
GW_DELAY	Groundwater delay time (days)	0–500	28
GWQMN	Threshold depth for shallow aquifer return flow (mm)	0–5000	1050
ESCO	Soil evaporation compensation coefficient	0.01–1.0	0.68
SOL_AWC	Available water capacity of soil layer (relative)	−25% to + 25%	+ 19%
SOL_K	Saturated hydraulic conductivity (relative)	−25% to + 25%	−13%
SURLAG	Surface runoff lag coefficient	0.5–24	5.2
CH_K2	Effective hydraulic conductivity in main channel (mm/hr)	−0.01–150	32.4
REVAPMN	Threshold depth for revaporation from shallow aquifer (mm)	0–500	175

Model performance was assessed using Nash–Sutcliffe Efficiency (NSE), Percent Bias (PBIAS), coefficient of determination (R^2^), and Kling–Gupta Efficiency (KGE^36^), following the criteria of^37,38^. Results are given in Table 3.

**Table 3:** SWAT calibration and validation performance.

Metric	Calibration 2003–2015	Validation 2016–2022	Acceptable Threshold	Reference
NSE	0.76	0.71	> 0.50	Moriasi^37^
PBIAS (%)	− 8.4	+ 11.2	< ±25%	Moriasi^37^
R^2^	0.81	0.77	> 0.60	Moriasi^37^
KGE	0.74	0.69	> 0.50	Gupta et al.^36^

### CROPWAT 8.0 setup and crop water demand estimation

CROPWAT 8.0 was used as the estimation of crop water demand^39^. Daily IMD climate station data was used to calculate reference evapotranspiration (ET_o_) using the FAO-56 PenmanMonteith (PM) method^27^. Crop coefficients (Kc) were obtained as per FAO-56 and modified using field surveys of 2022-23 (Table 4). The calculation of effective rainfall was done according to the USDA Soil Conservation Service (SCS) method^22^. Soil inputs were derived from NBSS&LUP 1:50,000 maps: Vertisols (TASW 160–200 mm/m; root depth 0.9–1.2 m) and Alfisols (TASW 110–150 mm/m; root depth 0.6–0.9 m). The efficiency of conveyance (ηc = 0.72) and application on the field (ηa = 0.65) was borrowed through Narayanamoorthy^40^ and GoT (2019)^46^, resulting in an overall project efficiency in ηp = 0.47.

**Table 4:** Crop calendar and FAO-56 crop coefficients for the KLIS command area.

Crop	Season	Planting	Harvest	Kc_init	Kc_mid	Kc_late
Paddy (transplanted)	Kharif	Jul	Nov	1.10	1.20	0.90
Cotton	Kharif	Jun	Jan	0.35	1.15–1.20	0.50–0.55
Pulses (redgram)	Kharif/Rabi	Jul/Nov	Dec/Mar	0.40	1.05	0.30
Wheat/sorghum	Rabi	Nov	Mar	0.30	1.15	0.30–0.55
Vegetables (mixed)	Rabi/Pre-monsoon	Nov/Apr	Feb/Jun	0.60	1.05	0.90
Sunflower	Rabi/Pre-monsoon	Jan	May	0.35	1.10–1.15	0.35

### Monthly water-balance framework

The water-balance framework was implemented each month at the Hydrologic Response Unit (HRU) level, and subsequently, summed up to the KLIS command scale by area-weighted summation. The water balance written as:

1 $$\:\begin{array}{cccc}&\:{P}_{i,m}+{I}_{i,m}=E{T}_{c,i,m}+{Q}_{i,m}+{R}_{i,m}+{\Delta\:}{S}_{i,m} \end{array}$$

where $\:{P}_{i,m}$represents precipitation, $\:{I}_{i,m}$represents irrigation water applied, $\:E{T}_{c,i,m}$is crop evapotranspiration, $\:{Q}_{i,m}$is surface runoff, $\:{R}_{i,m}$is deep percolation or recharge, and $\:{\Delta\:}{S}_{i,m}$is the change in soil-water storage within the root zone. At the command scale, the same balance was obtained by aggregating the HRU-level values as area-weighted monthly totals:

2 $$\:\begin{array}{cccc}&\:{P}_{m}+{I}_{m}=E{T}_{c,m}+{Q}_{m}+{R}_{m}+{\Delta\:}{S}_{m} \end{array}$$

Reference evapotranspiration $\:\left(E{T}_{0}\right)$estimated using FAO-56 Penman Monteith equation that is universally applied in determining the irrigation-water requirement^27^:

3 $$\:\begin{array}{cccc}&\:E{T}_{0}=\frac{0.408{\Delta\:}({R}_{n}-G)+\gamma\:\left(\frac{900}{T+273}\right){u}_{2}({e}_{s}-{e}_{a})}{{\Delta\:}+\gamma\:(1+0.34{u}_{2})} \end{array}$$

In this equation, $\:{\Delta\:}$is the slope of the saturation vapour pressure curve $\:\left(kPa{\:}^{\circ\:}{C}^{-1}\right)$, $\:{R}_{n}$is net radiation $\:\left(MJ\:{m}^{-2}\:{d}^{-1}\right)$, * G*is soil heat flux, $\:\gamma\:$is the psychrometric constant, * T*is mean air temperature $\:\left.{(}^{\circ\:}C\right)$, $\:{u}_{2}$is wind speed at 2 m height $\:\left(m\:{s}^{-1}\right)$, and $\:{e}_{s}-{e}_{a}$represents the vapour pressure deficit $\:\left(kPa\right)$.

Crop evapotranspiration for each crop * c*and month * m*was computed by multiplying reference evapotranspiration with the relevant crop coefficient:

4 $$\:\begin{array}{cccc}&\:E{T}_{c,c,m}={K}_{c,c,m}\times\:E{T}_{0,m} \end{array}$$

where $\:{K}_{c,c,m}$denotes the crop coefficient corresponding to the crop type and growth stage during month * m*. Effective rainfall $\:\left({P}_{eff,m}\right)$was estimated using the USDA-SCS method [49], and the net irrigation water requirement was calculated as:

5 $$\:\begin{array}{cccc}&\:IW{R}_{m}=E{T}_{c,m}-{P}_{eff,m} \end{array}$$

where $\:IW{R}_{m}$is the net irrigation water requirement for month * m*. The net requirement was then adjusted to obtain the gross irrigation requirement, by including the conveyance and field-application efficiencies:

6 $$\:\begin{array}{cccc}&\:GI{R}_{m}=\frac{IW{R}_{m}}{{\eta\:}_{p}}=\frac{IW{R}_{m}}{{\eta\:}_{c}\times\:{\eta\:}_{a}} \end{array}$$

where $\:GI{R}_{m}$is the gross irrigation requirement, $\:{\eta\:}_{p}$is the overall project irrigation efficiency, $\:{\eta\:}_{c}$is canal conveyance efficiency, and $\:{\eta\:}_{a}$is field-application efficiency.

Monthly requirements (supply -demand) in the command scale was determined by :

7 $$\:\begin{array}{cccc}&\:{D}_{m}=GI{R}_{m}+DW{R}_{m}-C{S}_{m}-GW{S}_{m}-R{F}_{m} \end{array}$$

where $\:{D}_{m}$is the monthly deficit, $\:DW{R}_{m}$is domestic water requirement, $\:C{S}_{m}$is canal supply, $\:GW{S}_{m}$is groundwater supply, and $\:R{F}_{m}$is return flow. All volume-based terms were expressed in million cubic metres $\:\left(MCM\:or\:M{m}^{3}\right)$. A positive value of $\:{D}_{m}$indicates unmet demand, whereas a negative value indicates surplus water availability. The runoff $\:\left({Q}_{m}\right)$, recharge $\:\left({R}_{m}\right)$, and soil-water storage change $\:\left({\Delta\:}{S}_{m}\right)$were obtained from SWAT HRU outputs and aggregated to the command scale using area-weighted summation.

### Software and post-processing

Spatial processing and DEM analysis ArcGIS Desktop 10.8.2^29^ in ArcHydro, ArcSWAT 2012^30^. SWAT simulation: SWAT 2012 Revision 700^33^. Calibration/uncertainty: SWAT-CUP v5.3.7^35^. Crop-water estimation CROPWAT 8.0^39^. After-processing, statistical analysis and visualisation of supply and demand: MATLAB R2023a (The MathWorks Inc., 2023)^48^ and Microsoft Excel 2019.

## Results

### Spatial characteristics of the KLIS command

**Fig. 3:**
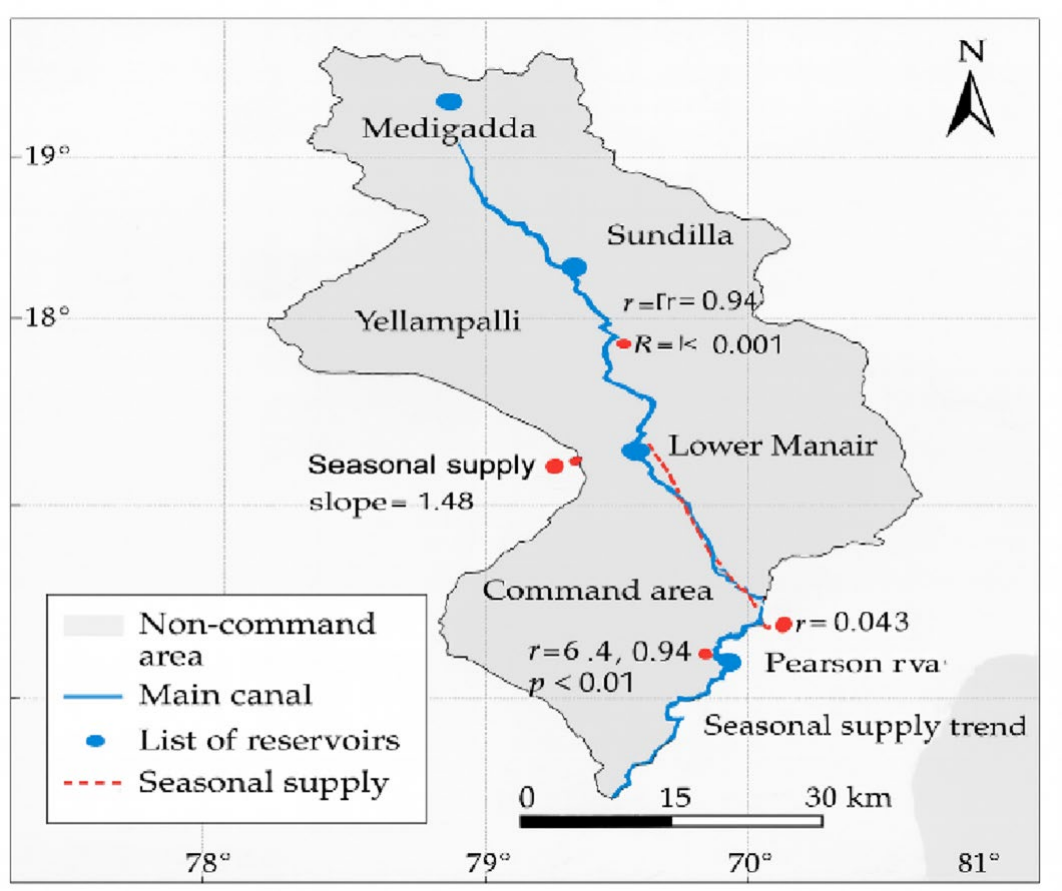
KLIS Location Map showing the Godavari River, key reservoirs (Medigadda, Sundilla, Yellampalli, Mid Manair, Lower Manair), the main canal alignment, command and non-command area boundaries, latitude–longitude graticule, and scale bar.

The general position of the Kaleshwaram Lift Irrigation Scheme (KLIS) is indicated in Fig. 3 which also defines the irrigated command in terms of non-command areas. The scheme lies along the rivers Godavari, which runs between the Lower Manair Reservoir and the Medigadda barrage in the south and north respectively 18°–19° N latitude and 78°–81° E northsouth and an area of about 7620 km^2^ of gross command. The primary canal spine has five consecutive lift stages, Medigadda, Sundilla, Yellampalli, Mid Manair and Lower Manair, which serve as hydraulic control points that control the timing and contents of releases to downstream distributaries.

Another structure that plays a significant role in the performance of irrigation is the spatial gradient in hydraulic accessibility. Head region areas are supplied more reliably and timely compared to tail end regions towards Lower Manair which have lower reliability of delivering and short supply periods. This spatial heterogeneity shows up in the pattern of cropping and water-use intensity recorded and it is an important explanatory variable to the spatially uneven distribution of irrigation deficits observed in the supply demand analysis .

### Terrain and hydrological connectivity

**Fig. 4:**
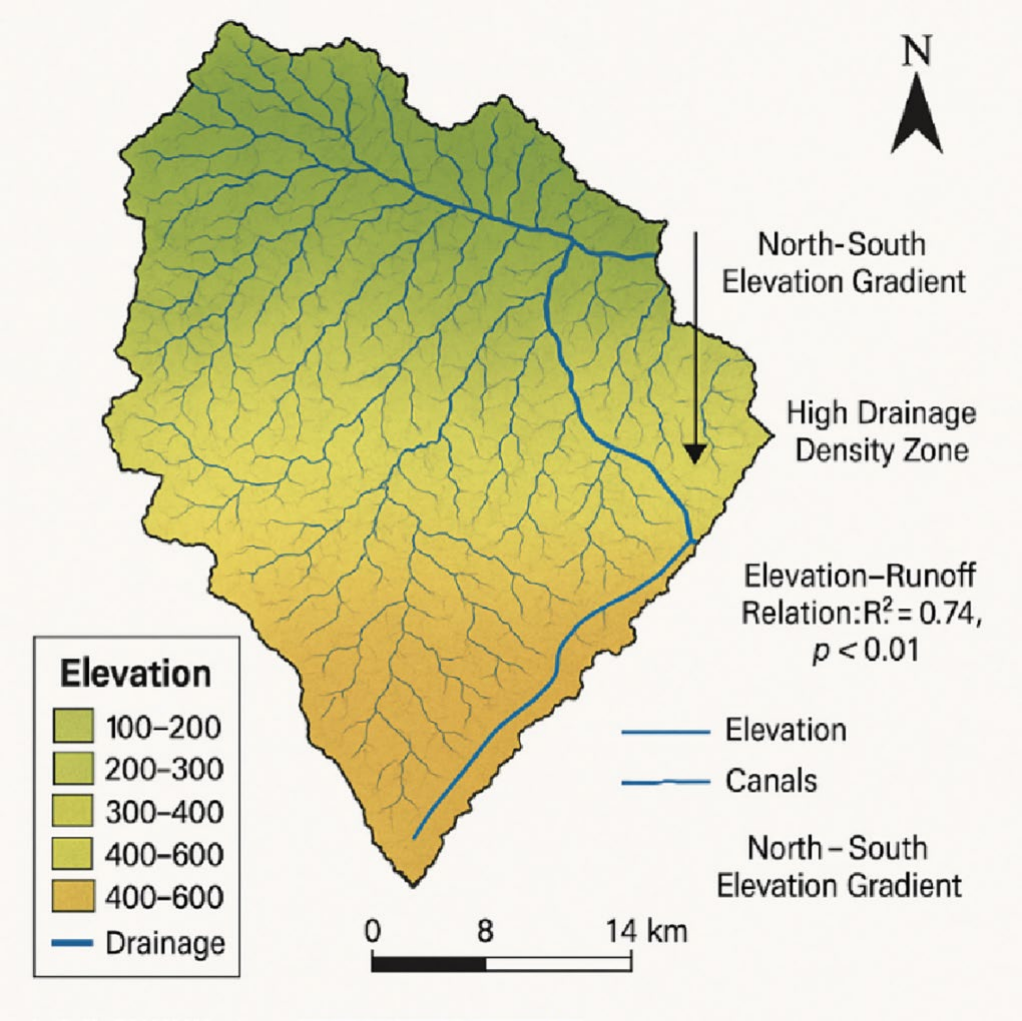
DEM and Drainage/Canal Network of KLIS Command showing the north–south elevation gradient, dendritic drainage pattern, and alignment of primary conveyance canals.

Figure 4 shows the digital elevation model (DEM) of KLIS command area superimposed with the natural drainage network and canal network. Its topography has a sharp north south elevation range: upper northern and north-eastern catchments are over 400 m in the upper part of the command and falling to 200–300 m in central area and below 200 m in the south. This gradient regulates overland flow production, soil moisture redistribution, and groundwater recharge potential- those which have direct impact in the monthly water-balance components.

The drainage system seen in Fig. 4 is the dendritic one which consists of natural tributaries leading to the Godavari River. Having a high drainage density in the central and the northern areas of the command is what identifies the area as being high run off responsive during the monsoon which is in line with the period of the maximum run off of 55–60 mm experienced in July–August as indicated in Table 6. The fact that the main lines of the canal coincide with constant heights plates justify the fact that KLIS infrastructure had been designed to take advantage of natural topographic gradients, keeping the pumping head across lift stages as low, and the conveyance energy level as low as possible and assisting in a reasonably even distribution to distributaries.

### Land use and cropping patterns

**Fig. 5:**
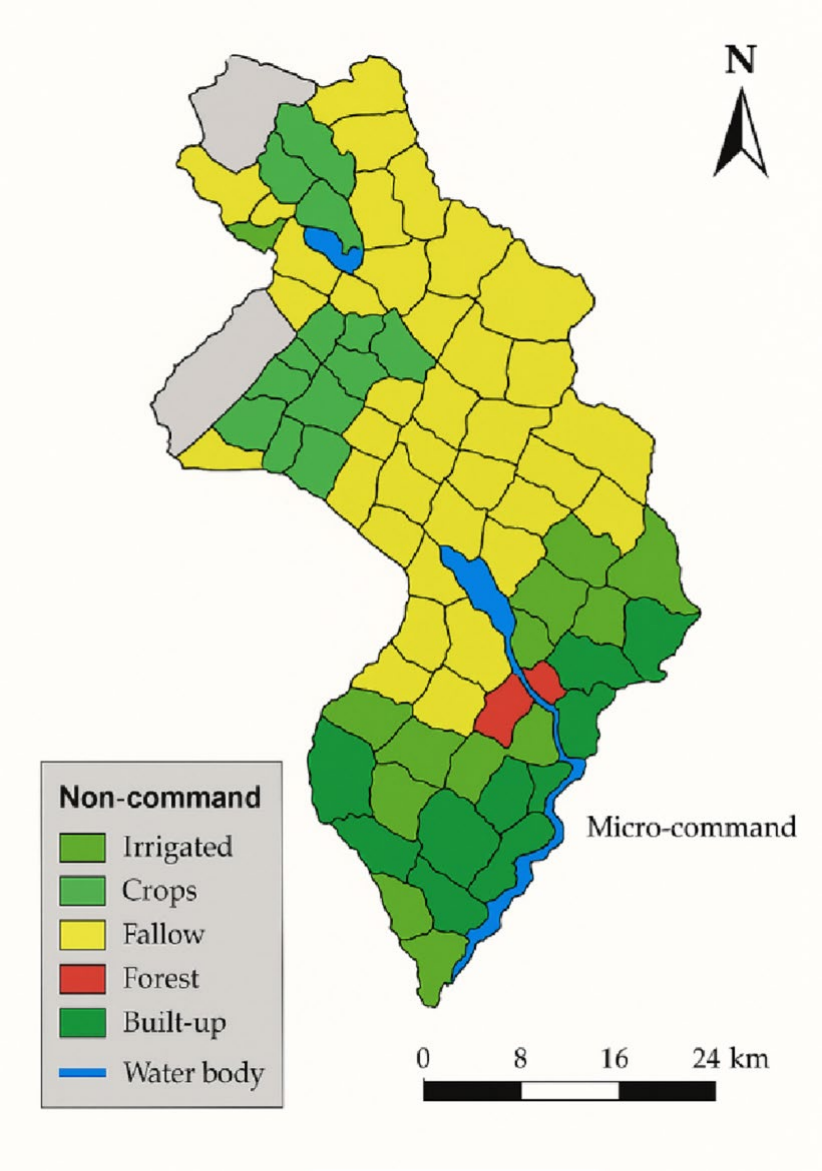
Land use and cropping system map of the KLIS command area.

As shown in Fig. 5, land use and the types of crops cover the entire area of the KLIS command area; in the micro-command areas of central and south, the irrigated agriculture is the dominant type. To facilitate understanding, the irrigable area between Sundilla and Yellampalli lift stages is called the central micro-command area (approximately 78–80° E, 18.5–19° N), while the south micro-command area encompasses the lower-reach zone from Yellampalli to the Lower Manair reservoir (approximately 18–18.5° N); the two districts include about 58% of the total command area, and are best supplied by the canals since they are close to large distribution centres.

The low and central command areas (74,500 ha; Table 5) dominate Paddy, with the raised canal releases being comparatively steady and providing high-confidence irrigation during the Kharif season. The fact that it has a high mid-season crop coefficient (Kc_mid = 1.20; Table 5) renders Paddy the sole largest source of seasonal crop water demand in the water deficit. The moderately elevated lands in the mid-command have a greater presence of cotton (68, 200 ha) and maize (32, 800 ha), as they require less and are more adaptable to water needs when compared to those of paddy. Tail-end areas are concentrated on groundnut, chillies and mixed vegetables, with less assured canal supply, and whose farmers rely on residual soil moisture or rainfall.

Sugarcane and perennial horticulture are planted in areas near distributaries and lift station (28,700 ha and 22,300 ha, respectively), with sufficient water supply to occupy their long crop cycles (300–360 days to sugarcane) and high cumulative evapotranspiration requirements. The presence of fallow and current-fallow land (15,900 ha) in the northern area and fringe area shows that some land cannot be viable to intensive cultivation due to soil constraints, topography location or distributary access, and also reflect the areas which could be available to future food production should reliability of supply increase.

**Table 5:** Crop types, crop coefficient (Kc) values by growth stage, crop duration, and command area under KLIS Note: Kc values are based on FAO-56 standards (^27^, adjusted for local agro-climatic conditions. Crop coefficients represent: Initial = establishment phase; Mid-Season = maximum canopy cover and transpiration; Late Season = maturation and senescence.

Crop type	Kc (initial)	Kc (mid-season)	Kc (late season)	Crop duration (days)	Command area (ha)
Paddy (Kharif)	1.05	1.20	0.90	120–135	74,500
Maize	0.30	1.20	0.60	100–110	32,800
Cotton	0.35	1.15	0.65	160–180	68,200
Groundnut	0.40	1.10	0.70	110–120	18,400
Chillies	0.50	1.05	0.90	150–180	12,600
Vegetables (mixed)	0.40	1.00	0.80	90–120	9200
Sugarcane	0.40	1.20	1.10	300–360	28,700
Horticulture/plantation	0.50	0.95	0.75	Perennial	22,300
Fallow/current fallow	–	–	–	–	15,900

### Seasonal rainfall and atmospheric water demand

Table 6 shows the average (2000–2023- 24 years; *n* = 24 years) monthly water balance of the KLIS command. The variables are defined as below: Rainfall (P) = precipitation that was measured each month (mm); Reference Evapotranspiration (ET_o_) = potential evaporation of a reference grass surface with ample water, calculated using the FAO-56 Penman Monteith method; Crop Evapotranspiration (ETc) = actual crop water demand (ETc = Kc × ET_o_, area-weighted across all crops ); Runoff (Q) = surface runoff caused in rainfall exceeding the infiltration or absorption, predicted by a CN-based model; Recharge (R): deep percolation to groundwater; Initial and Final Soil Moisture (SM): volumetric soil water content at the beginning of each month (mm equivalent over the profile); Soil Moisture Change (ΔS) = Final SM − Initial SM; and Total Runoff = Q + R (total outflow from the soil water store). The water-balance equation is: P + I = ETc + Q + R + ΔS, where I= canal irrigation supply. The variability between years is great; the annual rainfall fluctuated between 712 mm (2002), up to 1284 mm (2020) over the record, with a coefficient of variation (CV) of 22.3, meaning that southwest monsoon is highly variable with time. Monthly ET_o_ had significantly less inter-annual variance (CV 8–12% in single months) in line with its sensitivity to temperature and solar radiation as opposed to monsoon processes.

Hydro- climatic record indicates the strong monsoonal signature that consists of three different hydrological phases:

1. Water-Deficit Phase (February–June): ET_o_ and ETc are greater than rainfall throughout the stage with the deficit continuing to increase with increasing temperatures and solar radiation to the pre-monsoon peak. The loss of soil moisture (ΔS) gradually reduces between − 3.0 mm (February) and − 15.0 mm (May) with crops competing with water in the shallow soil. By April–May, ET_o_ has 150–170 mm and only 25–50 mm of rainfall giving a 100–120 mm difference in atmospheric requirement at the month end that has to be supplied by the canals. The monthly rainfall (distributed with a standard deviation of 8 mm in January) shows a values distribution of up to 28 mm in June showing moderate but significant inter-annual variability of the time when the rainy season starts.
2. Water-Surplus Phase (July–September): the change in the hydrological balance upon the arrival of the monsoon is very abrupt. The water year is finally surpassed by July and August (210–230 mm- ETc 150–145 mm) which result in peak runoff (55–60 mm) and recharge (28–32 mm), and a positive ΔS (+ 20 to + 25 mm/month). The mean monthly rainfalls during this period are of 38–45 mm indicating highly inter-annual variation in the intensity of monsoons. Recharge that is formed during this time period plays a vital role in groundwater replenishment; compared to direct rainfall percolation, the correlation between recharge and canal seepage (Table 6), 0.56 shows that there is a significant additional contribution to aquifer recharge due to seepage of the canals.
3. Post-Monsoon Transition (October–January): With the removal of monsoon, amount of rainfall decreases at a high rate. and ET_o_ again exceeds precipitation. The decline in soil moisture negatively occurs at − 5.0 to − 8.0 mm/month up to November, leaving a storage at rest which underpins early Rabi crop planting. Soil moisture reaches a level of 80–98 mm by December–January, the first condition of the second pre-monsoon deficit cycle.

The annual ΔS of + 18.0 mm is positively valued, and deserves particular comment as, perhaps, it seems contrary to intuition within a water-deficit system. This value corresponds to the slow growth in the size of the KLIS command area over the study period: the total irrigated area in the early years of operation (2000-2007) was much less than the design capacity, actual ETc was less than the average of the long run, and monsoon recharge was (relatively) free, resulting in net positive 1 S which is concluded in the multi-year average. The 18.0 mm change is about 1.2% of the nominal water-holding capacity of soil and it lies within the limits of the ± 5% of uncertainty of the water-balance model; it is not to be construed as a trend in a direction of unsustainable accumulation of soil moisture.

**Table 6:** Multi-year average monthly water balance of KLIS command (mm) [Averaging period: 2000–2023; *n* = 24 years] Variable definitions: ET_o_, reference evapotranspiration; ETc, crop evapotranspiration; SM, soil moisture; ΔS, change in soil moisture (Final SM − Initial SM); Total Runoff, surface runoff + recharge. All values in mm. Mass-balance residuals range from 0.4% (August) to 4.8% (April), mean = 2.1%, confirming internal consistency.

Month	Rainfall	ET_o_	ETc	Runoff	Recharge	Initial SM	Final SM	ΔS	Total runoff
Jan	7.0	80.0	70.0	0.0	5.0	80.0	85.0	5.0	5.0
Feb	10.0	95.0	85.0	0.0	6.0	85.0	82.0	−3.0	6.0
Mar	15.0	120.0	110.0	1.0	7.0	82.0	76.0	−6.0	8.0
Apr	25.0	150.0	140.0	3.0	9.0	76.0	66.0	−10.0	12.0
May	50.0	170.0	160.0	8.0	11.0	66.0	51.0	−15.0	19.0
Jun	120.0	160.0	155.0	25.0	20.0	51.0	61.0	10.0	45.0
Jul	210.0	150.0	150.0	55.0	30.0	61.0	86.0	25.0	85.0
Aug	230.0	140.0	145.0	60.0	32.0	86.0	106.0	20.0	92.0
Sep	180.0	130.0	135.0	45.0	28.0	106.0	116.0	10.0	73.0
Oct	90.0	110.0	115.0	15.0	15.0	116.0	111.0	−5.0	30.0
Nov	30.0	90.0	95.0	3.0	8.0	111.0	103.0	−8.0	11.0
Dec	10.0	80.0	80.0	1.0	6.0	103.0	98.0	−5.0	7.0
Annual	977.0	1,475.0	1,440.0	216.0	167.0	—	—	18.0	383.0

### Monthly crop water requirement and canal supply

**Fig. 6:**
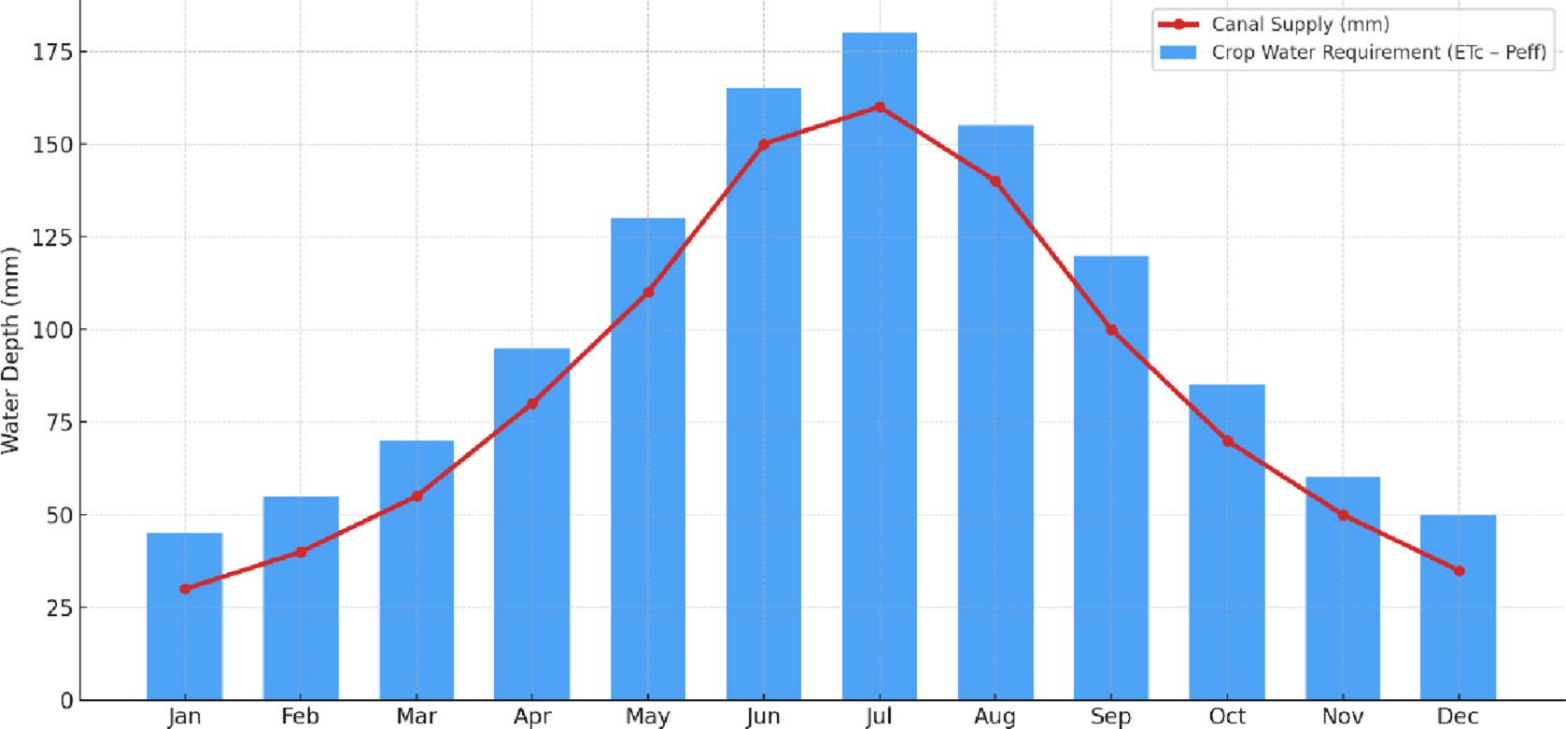
Monthly crop water requirement (CWR) and Canal supply (KLIS Command, 2000–2023). Shaded bands represent 95% confidence intervals derived from inter-annual variability. CWR = ETc − effective rainfall (Peff).

Figure 6 presents a comparison of monthly crop water requirement (CWR) as ETc and Peff (the difference between ETc and effective rainfall) i.e., net irrigation demand that needs to be met through canals versus the gross canal releases actually achieved. The Canal supply data are the releases of the main canal headworks and major distributary gauging stations of active discharge registers prepared by the Telangana Irrigation Development Corporation (TIDC) for 2000–2023; they are measured values and not design estimates. The interpolation of upstream reservoir release records based on the values provided incomplete records (smaller than 5% of the dataset) was carried out by using a calibrated routing coefficient.

The 95% confidence intervals (CI) displayed in Fig. 6 are calculated based on the inter-annual variability over the 24 years record. The narrowest CWR confidence bands are found in July-August (± 8–12 mm), and this is because, monsoon-season crop water requirements are the most consistent due to the unchanging temperature and radiation; and the broadest in May–June (± 18–24 mm), due to variation in the onset of the monsoon rainfall across years. Canal supply CIs are broadest in June-July (± 15–20 mm) due to the variability of the timing of inflow to reservoir and pre-monsoon reservoir levels.

CWR rises continually after February as ET_o_ rises with rise in temperatures and solar radiation to peaks of 150–180 mm in June–July, early Kharif establishment phase when the evaporative demand of the atmosphere was greatest and the canopy of the crops planted had not grown. An annual average and quantitatively significant difference in CWR supply as compared to canal supply is observed throughout the year but is even more acute (15–25 %) during the critical months of June–July. This long-standing gap may be attributed to three operating and infrastructural reasons:

- Stabilisation of the reservoir’s prerequisite: The reservoir level at Sundilla and Yellampalli is prerequisite: Before full-scale discharge of a distributary can be commenced, the level of reservoirs shall be stabilised beyond minimum operating levels. This limitation normally causes the beginning of Kharif canal supply to be 2–3 weeks later compared with the peak crop water demand.
- Staggered head-reach prioritisation: TIDC operating rules direct early releases to upstream head-reach distributaries to fill conveyance structures and satisfy demand before directing flow to lower command reduced the flow available to the lower command during the early Kharif season.
- Conveyance and seepage losses: The average conveyance losses through the multi-lift system is 12–15% of the diverted volume, which further decreases the amount of water to the farm outlets compared to the headworks discharges.

By late August CWR is lower as monsoon rainfall increases and crops enter the reproductive and maturation phases with lower water demand; canal supply is also lower as the operations in the reservoirs change to conservation. Even at this time, there remains a residual supply deficit, and this testifies to the constitutive dependence of the working of the KLIS on accurately timed canal work all through the Kharif season.

### Monthly water balance components

**Fig. 7:**
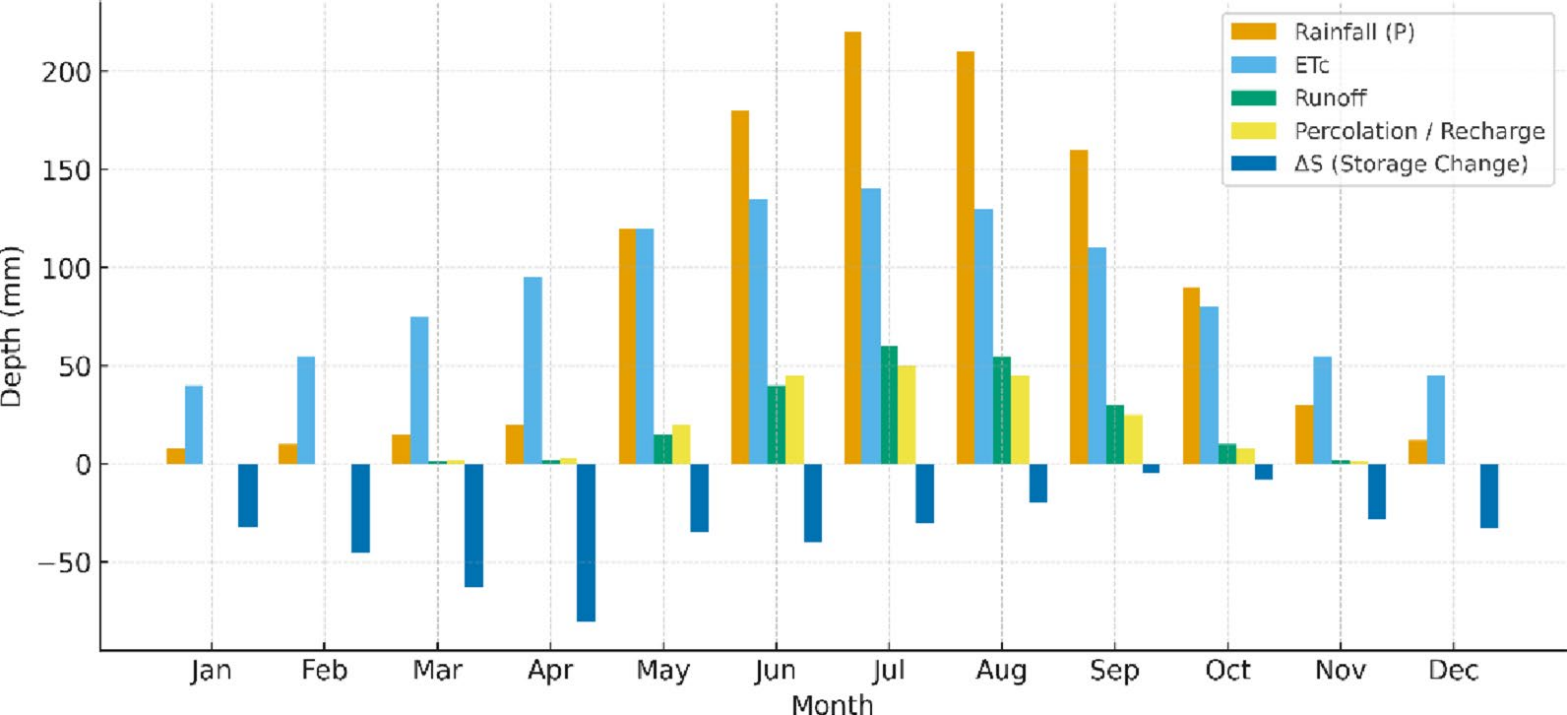
Monthly water balance components for the KLIS Command (24-year average, 2000–2023): monthly rainfall (P), crop evapotranspiration (ETc), runoff (Q), recharge (R), and soil moisture change (ΔS).

Figure 7 presents a unified perspective of the monthly water balance elements of the KLIS command that summarises the data in Table 6 and displays it in a graphical form (representing seasonal water behaviours). The figure underscores the time-based concentration of water available and how seasonal surpluses and deficits are produced.

A significant increase in rainfall (120–230 mm/month) is recorded during the monsoon (June–September) when the water year experiences a peak runoff (25–60 mm/month) and recharge (20–32 mm/month) the first time in the water year compared to ETc (135–155 mm/month). The total outflow (Q + *R* = 45–92 mm/ month) of this period makes up about 77% of the total amount of runoff, which is 383 mm annually, which means that the monsoon comprises the main contributor to surface and ground water renewal. Large positive ΔS values (+ 10 + 25 mm/month) in the period July-September show that the soil profile is actively filling with moisture to give us a post-monsoon crop-demand carry-over store.

During the pre-monsoon (February–June) ETc is greater than rainfall by 60–130 mm/month and essentially no runoff or significant recharge occurs. Unfavourable changes in the ΔS (0 or − 3 to − 15 mm/month) indicate drying out of the soil in a systematic manner - that the 85 mm of soil moisture in February fell to 51 mm by the end of May, and it lost 34 mm (cumulative depletion). This loss should be compensated by irrigation of canals, which measures the minimum irrigation need without consideration of weather conditions.

During the post-monsoon transition (October–January) it once again becomes positive, with ETc surpassing rainfall (by 40–70 mm/month), and small negative %S values (− 5–8 mm/month) represent remaining soil moisture drawdown. Groundwater replenishment decreases dramatically since runoff and recharge decreases. Carryover of monsoon water offers the ground a relatively high level of absolute soil moisture (103–111 mm) which allows establishment of Rabi crops to take place to a significant contest to full canal releases.

All these water balance processes underpin the underlying seasonal asymmetry of the KLIS hydrological regime about 76% of the annual rainfall is received during June–September, whereas crop water demand continues across all twelve months., whereas the total crop water demand (ETc = 1440 mm/annually) is received in all twelve months, which places an inherent structural deficit that has to be filled by irrigation infrastructure. It is this asymmetry that is the leading contributor to the 947 Mm^3^/year net writ-up of irrigation deficit that is seen in Table 7.

### Stage-wise irrigation demand and supply

**Fig. 8:**
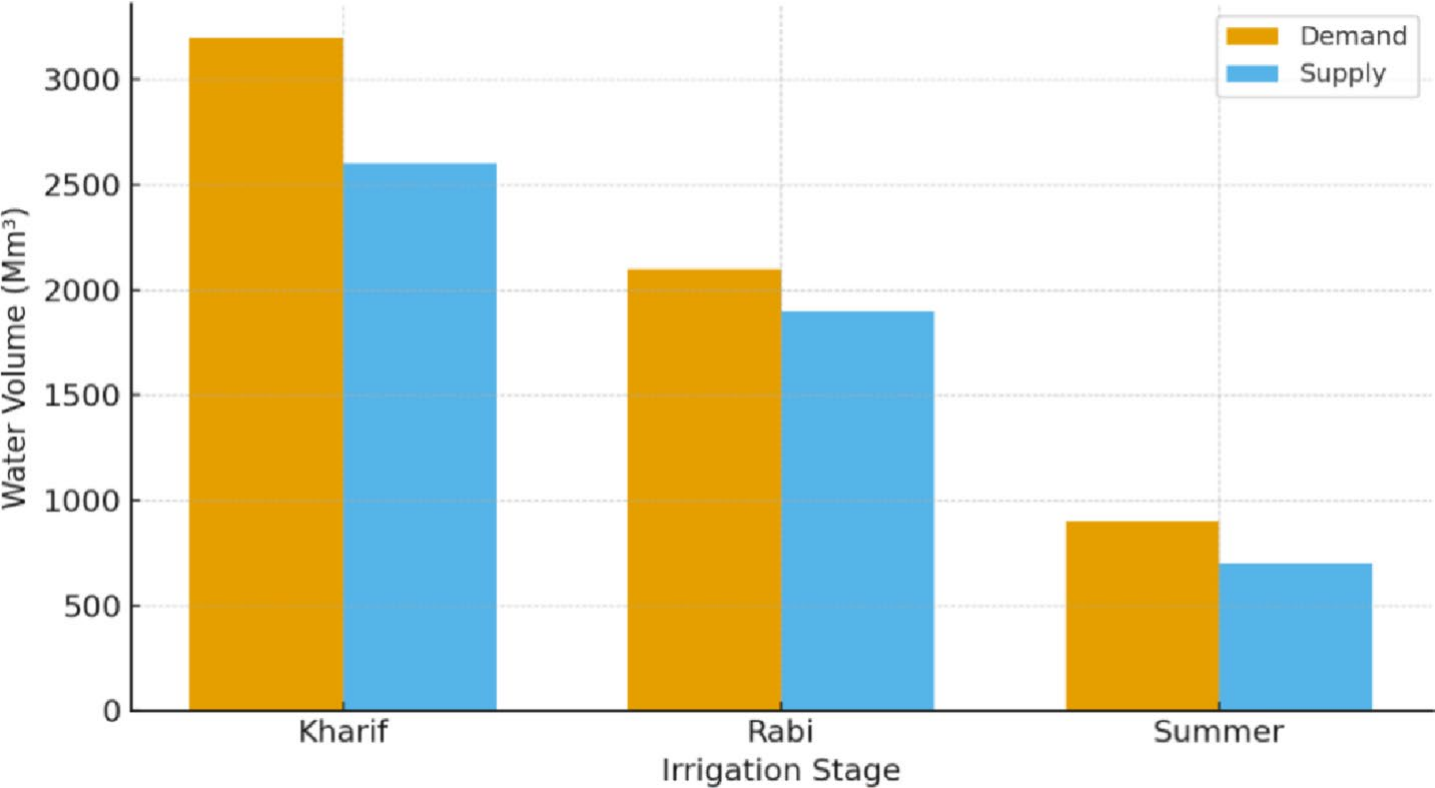
Stage-wise irrigation water demand vs. canal supply across Kharif, Rabi, and Pre-monsoon (Summer) seasons (KLIS command). Values shown are seasonal totals (Mm^3^). Gross demand , total ETc; net irrigation demand, ETc − effective rainfall.

Figure 8 sums up the irrigation water demand and actual canal supply in the three agricultural seasons Kharif (June–October), Rabi (November–February) and Pre-monsoon (Summer) (March–May) to give the seasonal perspective of the structural supply demand disequilibrium observed in the monthly analysis. The seasonal figures which are indicated against each season (ex. 3200 Mm^3^ of crop water used in Kharif) are the gross seasonal crop water demand (which means the sum of the total ETc of all crops in that season), however, this figure has not had the effects of effective rainfalls subtracted. Table 7 shows that the total of 1677 Mm^3^ of annual irrigation demand is actually the net annual demand in that, after deducting effective precipitation, the desired amount of irrigation water must be delivered by the canals and groundwater. Both the gross and the net values are measurements of vastly different quantities, and are incomparable; the apparent difference in one and the other can be perfectly explained by the deduction of the effective rainfall.

Kharif gross demand is about 3200 Mm^3^ as the limiting crop paddy at 74,500 ha with mid-season Kc 1.20 produces the greatest amount of evapotranspiration of the year when canopies reach their peak development and during the warm, humid weather. An estimated 45–50%. of the entire Kharif gross demand is taken up by Paddy alone. Sugarcane (28,700 ha; Kc_mid = 1.20) and cotton (68,200 ha; Kc_mid = 1.15) are the next largest contributors. The net canal supply required during Kharif is around 2600 Mm^3^ though despite the significant compensatory presence of the monsoon rainfall, the net gross demand is about half that amount of 2600 Mm^3^ meaning that the system heavily relies on the timely monsoon onset and the stored reservoir storage of the monsoon rainfall to establish the Kharif crop.

The escalated Kharif demand caused by the paddy farming also poses the question of less water-demanding alternatives. Crops like maize (Kc_mid = 1.20; crop duration 100–110 days) and sorghum need about 35–45% less seasonal water than paddy and yet give similar gross margins given the scenario in Telangana market. Pulses (pigeonpea, green gram) need a low seasonal water of between 350 mm and 450 mm as compared with 900 mm and 1200 mm of paddy, so planting such crops on some of the tail-end zoned paddy area could have a significant effect, as well as alleviating seasonal gap between supply and demand.

The gross seasonal demand is less during Rabi (November–February), when less water-intensive crops dominate (maize, groundnut, cotton), but canal supply is limited by lower post-monsoon reservoir storage levels. The associated Rabi shortfall of about 10% (in the gross demand) would mean that tail-end regions might have a less irrigated area or imposed crop substitution during years with below-normal monsoon storey. During the Summer season (March to May) there is simply not enough reservoir storage to store any remaining water, combined with maximum atmospheric demand, making canal supply a mere 70 to 75% of the seasonal demand, making Pre-monsoon (Summer) the hydraulically the most constrained season of the agricultural year and the season most heavily reliant on additional groundwater use.

### Monthly supply–demand balance and deficit

**Fig. 9:**
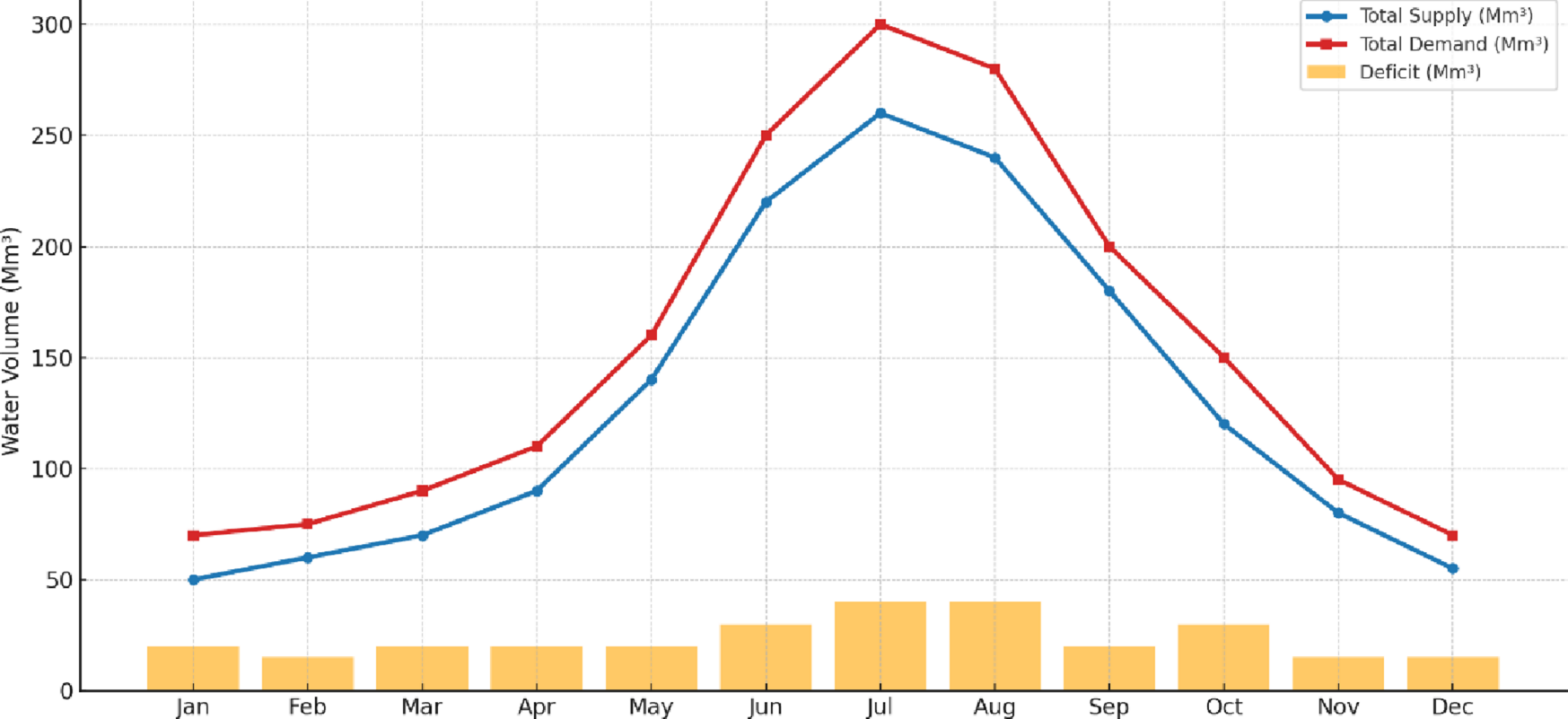
Monthly supply–demand volume and cumulative deficit for the KLIS command (Mm^3^).

The combined supply-demand balance of water in the agricultural year is shown in Fig. 9; Table 7 in monthly form, the KLIS command. The balance is characterised by three structural patterns:

Pattern 1: Continued deficit in all months: There is no single month in the 24-year record of supplies that indicates a supply excess. Total supply is out of demand even in the rainiest months of July (deficit = 7.3 Mm^3^) and August ( deficit = 12.3 Mm^3^) when it is at fullness. Such chronic shortage proves that the KLIS command is in chronic water shortage, which is impossible to overcome through operational optimisation.

Pattern 2: Severe pre-monsoon deficits: March–May (102–127 Mm^3^/month) take the place as the largest monthly deficits as the maximal atmospheric evaporative demand is more than the lowest canal release capacities of the year. These three months would add up to 351.8 Mm^3^ (37.1%) of the 947 Mm^3^ annual deficit even though it takes up 25% of the calendar year. The annual deficit is obtained by summing monthly unmet demand.

Pattern 3: Deficit even under deficits of the monsoons even with recharges: The small deficits (7.3 and 12.3 Mm^3^) in months July and August are enough to diagnose that even with maximum monsoon rainfalls the operational lag of the canal build-up process and the large initial water demand of transplanted paddy yield a net deficit. The sequentially of Kharif crop planting causes this trend, as transplanting normally falls within the first two weeks of July, when the peak of water demand is recorded, at the point when canal systems are not fully running yet.

**Table 7:** Monthly available supply vs. demand and deficit, KLIS command (Mm^3^) Groundwater and return-flow supply estimated from TSGWD shallow-aquifer extraction records and canal seepage return-flow coefficients of 8–12% applied to surface deliveries (Kumar et al.,2008)^47^. Treated as approximately constant at 5.0 Mm^3^/month based on < 15% inter-monthly variability in extraction records; sensitivity test (± 20% variation) changes annual deficit by < 5%. .

Month	Surface-water supply (Mm^3^)	Groundwater/return-flow (Mm^3^)^a^	Total supply (Mm^3^)	Total demand (Mm^3^)	Deficit (Mm^3^)	Cumulative deficit (Mm^3^)
Jan	40.0	5.0	45.0	137.3	92.3	92.3
Feb	50.0	5.0	55.0	152.1	97.1	189.4
Mar	60.0	5.0	65.0	167.3	102.3	291.7
Apr	70.0	5.0	75.0	197.2	122.2	413.9
May	80.0	5.0	85.0	212.3	127.3	541.2
Jun	75.0	5.0	80.0	122.2	42.2	583.4
Jul	50.0	5.0	55.0	62.3	7.3	590.7
Aug	45.0	5.0	50.0	62.3	12.3	603.0
Sep	40.0	5.0	45.0	92.2	47.2	650.2
Oct	50.0	5.0	55.0	152.3	97.3	747.5
Nov	60.0	5.0	65.0	167.2	102.2	849.7
Dec	50.0	5.0	55.0	152.3	97.3	947.0
Annual	670.0	60.0	730.0	1,677.0	947.0	947.0

The contribution of 5.0 Mm^3^/ month (60 Mm^3^/year) to the groundwater and return-flow as indicated in Table 7.. Two sources were used to estimate this value: (i) an average of all the records of shallow-aquifer extraction during the years 2000–2023 in the Telangana State Groundwater Department (TSGWD) monitoring network within the command area; and (ii) canal seepage of 8–12% of the monthly surface-water deliveries which was used to estimate it. This value reflects a conservative average of observed extraction and seepage driven ridden flow. The extraction total recorded between the months was less than 15% which is sufficient to treat the term as almost constant between the months. In order to evaluate the sensitivity of the conclusions to this assumption, the change in the groundwater term was given a scope of ± 20%: this changed the calculated amount of the deficit per year by a margin of less than 50 Mm^3^ (5.3%), implying that the major results are not sensitive to changes in this parameter.

Given that no month shows a supply surplus, the long-term sustainability of agricultural activity in the KLIS command area remains a critical concern. There are various resilience mechanisms developed by the farmers to meet the persistent deficit situation: (i) voluntary crop substitution naming paddy with maize, pulses or groundnut in tail-end command areas when canal supply is unpredictable; (ii) use of the residual monsoon soil moisture to meet the early post monsoon demand especially those required to establish the Rabi crop; (iii) supplemental use of private borewells or shallow groundwater sources. Although these coping strategies support continued agricultural activity, they may reduce yield potential, increase income instability, and, in the case of groundwater use, create long-term risks of aquifer depletion.

The minor yet noticeable July–August deficits are due to the chronological character of the monsoon onset and Kharif crop set up. The paddy is transplanted in the 74,500 ha area in the first 23 weeks of July, and results in maximum initial water demand (ETc ≈ 150 mm/month) at the time when the canal system is in its state of operational build-up. Sundilla and Yellampalli reservoirs is not usually full of distributary discharge until mid-July, presenting a lag during which the distribution crops will have to be nourished exclusively by rainfall. Even this small shortage increases significantly in magnitude in years when the onset of the monsoon is late or of lesser intensity, resulting in delays in transplanting and crop stress at the very weakest stage of establishment.

The 947 Mm^3^ yearly deficit is a structural imbalance, which needs short-term, operational interventions in addition to long-term strategic interventions. Domestic and industrial shifts do not significantly affect the ratio because irrigation has a share of about 99% of the total water needs hence the following prioritised intervention structure is recommended as evidenced by the scenarios found in the literature:

3 years supply side: stagger the canal arrangement in June-July to pre-load across to tail-end locations during pre-monsoon periods; initiate rule-curve changes to bunds  10–15% of pre-monsoon reservoir buffer stores; enhance specific conjunctive groundwater manufacture on rendezvous tail-end reaches. Biggs^41^ showed that staggered scheduling, alone, could decrease the peak-month shortages by 18–22% in similar lift irrigation systems.

- Short-term demand-side (3-5 years): Encourage Alternate Wetting and Drying (AWD) of paddy field tests on South Asia show that 15–30% water saved seasonally (with less than 5% yield loss) was achieved by field evidence^42^, because intermittent flooding reduces non-beneficial percolation and evaporation losses. Implement deficit irrigation methods of cotton and maize to those regions that get sufficient moisture buffering in the soils. Distribution losses can be eliminated by 8–12% with enhancement of farm-farm conveyance efficiency (field channel lining).
- Long-term structural (3-10 years): Promote cropping-pattern diversification with converting paddy on 15–20% of Kharif lands on deficit lands with less water-demanding crops (maize, pulses, vegetables). That, plus better scheduling, according to scenario modelling by^41^, would result in a reduction of annual net deficit to about 590–680 Mm^3^ − 28-38% without the need to install extra storage facilities, as lower crop water demand and improved release timing jointly reduce seasonal pressure on supply. Formalise allocation rules and ensure equitable tail-end allocation by developing command-area-level through water user associations (WUAs).

Concerning the long-term risks of conjunctive groundwater use : although the targeted groundwater extraction in tail-end areas will have the capacity to fill shortcomings in the short term, unregulated growth has high risks to sustainability. The correlation between recharge and release via canals (Table 6) gives a recharge-canal release value (rho = 0.56) and hence any diminution in the canal discharge would also diminish this recharge route and augment the depletion. The relevant regulatory authority is the Telangana Ground water (Regulation and Control of Development and Management) Act 2002 which requires registration of extraction structures and prohibition of new borewells in the identified over-exploited mandals. Proper enforcement of this Act coupled with input of groundwater monitoring systems by use of TSGWD piezometric network to monitor ground water every year is crucial to the avoidance of shift in reliance of supplemental to structural ground water dependence.

Within the socio-economic limitations to diversifying cropping patterns : being a dominant crop in the KLIS command, paddy would not be mainly hindered by the water availability, but rather the water availability is supported by a set of institutional components. One is that with the Government of India procurement scheme paddy is the beneficiary of a Minimum Support Price (MSP) that offers income security which other crops are unable to equal unless they too are procured. Second, due to the high rates of tenant-farming (good land has an estimated 35–45% of available operating acreage in Telangana), multi-year investments in non-perennial or infrastructure-intensive options are discouraged. Third, lack of a robust cold-chain and processing facilities against the perishable substitute products (vegetables, fruits) denies farmers the opportunity to experience a remunerative market structure. Some of the policy measures that may help to diversify would be: crop diversification bonuses which depend on water-use reduction; guaranteed MSP-equivalent purchases of identified substitute crops (pulses, oilseeds); subsidised drip and sprinkler irrigation of other crops; and policy development of farmer producer organisations (FPO) which would agglomerate supply and market risk.

A comprehensive Integrated Water Resource Management (IWRM) plan for the KLIS command area should incorporate the following key components: (i) real-time ET₀-linked reservoir operation scheduling to align releases with actual crop demand rather than fixed calendar dates; (ii) regulated conjunctive groundwater use with annual aquifer-level monitoring; (iii) seasonal crop planning aligned with supply-deficit projections, communicated to farmers before each season; (iv) on-farm conveyance efficiency investment targeting a 10–15% reduction in distribution losses; (v) tail-end equity mechanisms ensuring minimum supply commitments to the Lower Manair command even in deficit years; (vi) a monitoring and evaluation framework tracking monthly supply delivery, crop yield, and groundwater levels to enable adaptive management; and (vii) a structured participatory mechanism involving WUAs, TIDC, and TSGWD for inter-seasonal planning and conflict resolution.

This study has been informally released to the authorities at the Telangana Irrigation Development Corporation (TIDC) in a stakeholder consultation workshop. Early consultation signaled the interest in the conjunctive-use recommendations and the AWD recommendations; the adoption of the formulated policies will involve incorporation of the findings on the KLIS operating manual and state water policy of Telangana. Additional field information that would be most useful in analysing the information in future would involve: (i) continuous piezometric measurements of shallow aquifer throughout the command in place of the existing point-observation network; (ii) field level canal delivery measurements, during distributary and minor levels to lessen dependence on headworks information; (iii) in-situ measurement of soil moisture at representative farms along head- and tail-reach domains.

### Summary of statistical trend and significance tests

To evaluate formally the long-term trends and seasonal variations in the key hydrological variables, a set of statistical tests were used to the 24-year data (2000–2023). To test monotonic trend in annual time series (*n* = 24 annual observations) non-parametric Mann Kendall test was employed with Sen slope estimator to measure the strength of the trend; this test is most appropriate in hydrological records since it does not assume a distribution and it is also resistant to outliers and data gaps. To perform seasonal comparisons of variable results of the three agricultural seasons (Kharif, Rabi, Pre-monsoon ; *n* = 72 seasonal observations = 24 years × 3 seasons) a one-way ANOVA was considered when the variables maintained success of the Shapiro-wilk normality test (*p* > 0.05); a non-parametric statistic performed was the Kruskal–wallis H-test Pearson correlation (where both variables are normally distributed) or Spearman rank correlation (otherwise) were considered to be the pairwise associations between the variables of continuous types. The level of significance was 0.05. These findings are summarised in Table 8.

**Table 8:** Trend and significance tests for hydrological variables, KLIS command (2000–2023) Sample sizes: *n* = 24 annual observations for Mann–Kendall and Pearson/Spearman correlations; *n* = 72 seasonal observations (24 years × 3 seasons: Kharif, Rabi, Pre-monsoon ) for one-way ANOVA and Kruskal–Wallis tests. All variables were tested for normality using the Shapiro–Wilk test prior to test selection. Significance threshold: α = 0.05.

Variable	Test applied	Statistic	*p*-value	Trend/difference	Significance
Annual rainfall	Mann–Kendall	Z = − 0.84	0.401	No monotonic trend	Not significant
ET_o_ (Reference evapotranspiration)	Mann–Kendall	Z = + 2.11	0.034	Increasing trend (Sen’s slope = + 2.1 mm/yr)	Significant at 95%
Seasonal rainfall	One-way ANOVA	F = 19.4	< 0.001	Means differ across Kharif–Rabi–Summer	Highly significant
Crop-water requirement (CWR)	Kruskal–Wallis	H = 11.6	0.004	Seasonal medians differ	Significant
Irrigation deficit	One-way ANOVA	F = 4.21	0.018	Deficit varies among seasons	Significant
Runoff	Mann–Kendall	Z = + 0.59	0.553	No long-term trend	Not significant
Recharge	Mann–Kendall	Z = + 1.73	0.083	Weak increasing trend	Marginal significance (90%)
ET_O_ vs. CWR	Pearson correlation	*r* = 0.82	< 0.001	Strong positive relation	Highly significant
Rainfall vs. irrigation deficit	Pearson correlation	*r* = − 0.68	0.008	Higher rainfall reduces deficit	Significant
Recharge vs. canal releases	Spearman correlation	ρ = 0.56	0.021	Recharge increases with canal seepage	Significant

The statistical data proves the claim that the hydrological behaviour of the KLIS command area is regulated according to the systematic climatic and operational drivers and not by stochastic variability. A Mann–Kendall test shows that there is no significant long-term trend in rainfall in the annual amounts (Z = − 0.84 and *p* = 0.401), which is in line with historic evidence that no directional change has been observed in the monsoon-season totals on the Deccan Plateau^43^. The inter-annual changes in water supply can hence be significantly explained by the variations in the monsoon performance as opposed to a continuous pattern of precipitation.

In comparison, ET_o_ shows a statistically significant and positive increase that is sustained over time (Z = + 2.11, *p* = 0.034; Sen slope = + 2.1 mm/year), in line with reported warming and escalation of evaporative demand throughout semi-arid peninsular India^44^. This rate suggests a historical accumulating over 24 years the added evaporative demand at the command scale of circa 40-50 Mm^3^/year—the total supply margin in the system during Pre-monsoon -season. This increasing atmospheric demand is confirmed by the strong correlation between ET_o_ CWR (*r* = 0.82, *p* < 0.001) that fully espoused to increased crop water needs regardless of any cropped area expansion. These results have critical implications on the future sustainability of KLIS: assuming that the current trend of ET_o_ = +2.1 mm/year is maintained, the current deficit of 947 Mm^3^/year can be increased by 40–50 Mm^3^/decade without any alterations in the aspects of cropped area, crop mix, and operational strategy. This would mean that there can be a deficit of 1027−1047 Mm^3^ at a 20-year horizon- a 8–10% increment of the current benchmark. Adaptive modifications are thus necessary and encompass: (i) real-time monitoring of ET_o_ that is connected with scheduling algorithms of releases into the canal; (ii) the expansion of drought resistant, lower-kc types of crops; (iii) the gradual substitution of flood irrigation by drip and sprinkler systems so as to minimize losses in conveyance and application; and (iv) the systematic update of reservoir rule curves to.

The basic asymmetry of the climatic condition between Kharif, Rabi, and Pre-monsoon seasons which is the structural mechanism of the KLIS water deficit is quantified by the highly significant seasonal differences in rainfall (F = 19.4, *p* < 0.001), CWR (H = 11.6, *p* = 0.004), and irrigation deficit (F = 4.2). The low correlation between rainfall deficit and negative monsoon (*r* = − 0.68, *p* = 0.008) suggests that about 46% of the inter-annual variation in net irrigation deficit owes to monsoon performance - it can be determined that a year with under-normal Kharif rainfall is identified between June and implement pre-emptive conservation release and groundwater supplementation procedures. The presence of the positive correlation between recharge and release (r=0.56, *p* = 0.021) is an indicator that the activities of KLIS canals will have a significant effect in terms of the increase of the groundwater volume by seepage.

## Conclusion

This paper gives a seasonally-resolved evaluation of irrigation water demand and actual water supply in the Kaleshwaram Lift Irrigation Scheme (KLIS) command area by incorporating DEM-GIS preprocessing, SWAT-derived hydrology, CROPWAT-based crop-water requirement, and documented supply data of canal, groundwater and return-flow supply. The primary value of the paper is that the reliability of irrigation of a large semi-arid lift-irrigation command cannot be assessed using only the annual water availability but should be assessed using the monthly congruence of crop-water demand, effective rainfall, reservoir operation, canal releases and supplementary water sources.

The results show that the KLIS command has a long-term seasonal supply-demand imbalance whereby the water stress is the worst in the pre-monsoon’s when monsoon recharge is absent or inadequate, and the evapotranspiration is high. Under monsoon-favored farming, the occurrence of rainfall and the release of canals will always be a factor to consider, since the crops-setting and maximum development period should be supplied with trustworthy water supply. The study, therefore, emphasizes that the performance of KLIS depends not only on the presence of large-scale irrigation infrastructure but also the performance of this infrastructure in a relative sense to the seasonal crop calendars and the hydro-logical variability.

The policy implication of this writing is that the reduction of the deficit should be achieved by both the supply-side and the demand-side policies. At the supply side, by refining the curves of the reservoir rules, more responsive canal scheduling, better distributary-level regulation and planned conjunctive use of groundwater and completed water supply can help trim down the critical shortages of water per month. Crop planning management, reduction of water-intensive crops in micro-command structurally deficit zones; whenever agronomically viable, deficit irrigation, and enhanced water-use efficiency on farms, can enhance long-term sustainability of irrigation delivery on the demand side.

In a broader context, the research provides a generalizable model to evaluate the performance of irrigation in other semi-arid and monsoon-reliant commanded regions where in large infrastructural developments seasonal uncertainty exists, evapotranspiration is increasing, and where cropping is shifting. The research also notes that this analysis is constrained by the fact that dense field-level measurements are only available, the inability of the models to consider the conveyance efficiency, contribution of the return-flow, and more or less ignore the adaptive behaviour of individual farmers, including informal groundwater pumping, the switching of crops, and adjustment of the sowing-date. Future studies are required to develop the framework by experimenting with alternative reservoir-operation regulations, crop portfolio, irrigation technologies, and climate conditions in order to facilitate more robust command-area water management.

## Data Availability

The datasets used and/or analysed during the current study available from the corresponding author on reasonable request. All data generated or analysed during this study are included in this published article.
